# Acute on chronic liver failure: prognostic models and artificial intelligence applications

**DOI:** 10.1097/HC9.0000000000000095

**Published:** 2023-03-24

**Authors:** Phillip J. Gary, Amos Lal, Douglas A. Simonetto, Ognjen Gajic, Alice Gallo de Moraes

**Affiliations:** 1 Division of Pulmonary and Critical Care Medicine, Department of Internal Medicine, Mayo Clinic, Rochester, Minnesota, USA; 2 Multidisciplinary Epidemiology and Translational Research in Intensive Care Group, Mayo Clinic, Rochester, Minnesota, USA; 3 Division of Gastroenterology and Hepatology, Mayo Clinic College of Medicine and Science, Rochester, Minnesota, USA

## Abstract

Critically ill patients presenting with acute on chronic liver failure (ACLF) represent a particularly vulnerable population due to various considerations surrounding the syndrome definition, lack of robust prospective evaluation of outcomes, and allocation of resources such as organs for transplantation. Ninety-day mortality related to ACLF is high and patients who do leave the hospital are frequently readmitted. Artificial intelligence (AI), which encompasses various classical and modern machine learning techniques, natural language processing, and other methods of predictive, prognostic, probabilistic, and simulation modeling, has emerged as an effective tool in various areas of healthcare. These methods are now being leveraged to potentially minimize physician and provider cognitive load and impact both short-term and long-term patient outcomes. However, the enthusiasm is tempered by ethical considerations and a current lack of proven benefits. In addition to prognostic applications, AI models can likely help improve the understanding of various mechanisms of morbidity and mortality in ACLF. Their overall impact on patient-centered outcomes and countless other aspects of patient care remains unclear. In this review, we discuss various AI approaches being utilized in healthcare and discuss the recent and expected future impact of AI on patients with ACLF through prognostic modeling and AI-based approaches.

## Graphical abstract

**Figure:**
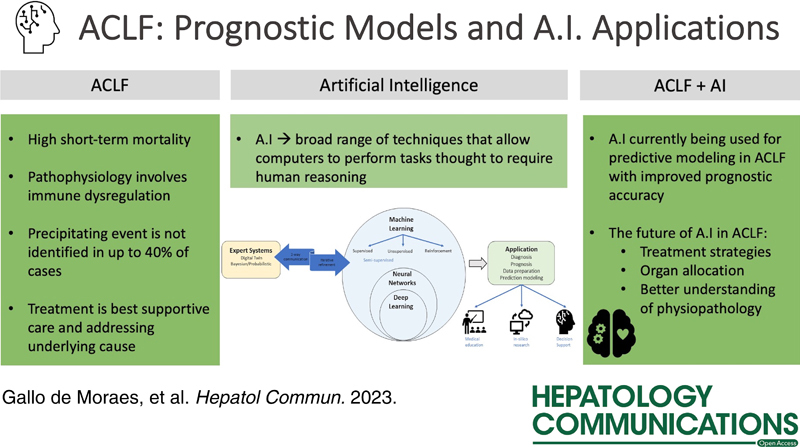

## INTRODUCTION

AI applications are being increasingly utilized in healthcare. Hepatology is no exception. Recently, there has been a significant application of artificial intelligence (AI) methodologies to hepatology including radiomics, histopathology, predictive and prognostic modeling, and estimation of posttransplant outcomes.^1^–^4^ To date, however, little data exist regarding AI applications specifically for critically ill patients with chronic liver disease the majority of whom meet diagnostic criteria for ACLF. This review seeks to outline ACLF as a syndrome, discuss current prognostic models for ACLF, define AI applications that are being used in healthcare, and discuss both current and potential AI applications in ACLF specifically.

### ACLF

ACLF is a recently recognized syndrome and is associated with increased short-term mortality.^5^,^6^ ACLF represents a syndrome in which there is systemic inflammation, in the setting of an inciting event, leading to the development of 1 or more organ failures including, but not limited to, acute decompensation of cirrhosis.^5^,^7^,^8^ The pathophysiology of ACLF is still unknown but immune dysregulation, similar to that seen in sepsis, with severe inflammation and oxidative stress have been implicated.^9^

In up to 40% of patients presenting with ACLF, no precipitating event is identified. Infection, in particular spontaneous bacterial peritonitis and pneumonia, has been deemed a common cause of ACLF and has been independently associated with mortality.^5^,^10^ Other proposed precipitants include gastrointestinal bleeding, alcohol-associated hepatitis, drug-induced hepatitis, acute or reactivation of viral hepatitis, and less commonly, surgery, TIPS creation, or trauma.^5^,^7^,^11^,^12^ Identification of and intervention targeted toward the underlying etiology is essential and prospective data have demonstrated that ACLF is potentially reversible especially when considering the ACLF grade at presentation.^13^ Given the dynamic nature of ACLF, aggressive critical care focusing on identifying and addressing the associated organ failure or failures, is recommended for the first 3–7 days, while considering prognosis based on the patient’s progression or improvement.^11^ Current prognostic scores as highlighted below, were developed predominantly with traditional statistical methods and are limited in their capability of providing actionable or meaningful data in the acute phase of critical illness.

Currently, multiple definitions exist for ACLF as outlined by the European Association for the Study of the Liver—Chronic Liver Failure (EASL-CLIF), North American Consortium for the Study of End-stage Liver Disease (NACSELD), and the Asian Pacific Association for the Study of the Liver (APASL) (Table 1).^16^ These definitions cohort ACLF patients based on geographic distribution and etiology.

**TABLE 1:** Definitions of ACLF by leading international consortia

Group	EASL-CLIF^5^	NACSELD^14^	APASL (AARC)^15^
Criteria	Hospitalized for AD cirrhosis, CLIF-SOFA ≥1, 28-d mortality >15% due to AD of cirrhosis+prior AD	Hospitalized with infection or develop infection while hospitalized, ≥2 OFs	Hospitalized for AD chronic liver disease (±cirrhosis), acute jaundice, and coagulopathy±HE
Exclusions	Scheduled procedure or treatment, HCC outside Milan criteria, severe chronic extrahepatic disease, HIV, immunosuppressives	Outpatient, HIV history, prior transplant, advanced malignancy	Bacterial infection or prior AD

Abbreviations: AARC, APASL ACLF Research Consortium; ACLF, acute on chronic liver failure; AD, acute decompensation; APASL, Asian Pacific Association for the Study of the Liver; EASL-CLIF, European Association for the Study of the Liver—Chronic Liver Failure; NACSELD, North American Consortium for the Study of End-Stage Liver Disease; OF, organ failure.

Adapted from Kumar et al^7^ and Olson.^11^

The EASL-CLIF definition was developed using a cohort of patients with predominantly alcohol-induced liver disease and utilized a Chronic Liver Failure—Sequential Organ Failure Assessment (CLIF-SOFA) score, which was a prespecified modified Sequential Organ Failure Assessment (SOFA) score specifically developed for the purposes of identifying and grading ACLF.^5^,^17^ The NACSELD definition was adapted from a previously defined infection-related ACLF score and subsequently validated in both infected and noninfected patients.^11^,^14^ The APASL definition was developed using a cohort of patients with predominantly HBV-related liver disease and has been recently validated in a non-Asian/Pacific Islander population.^14^,^15^,^18^,^19^

Prior prognostic scores used to estimate the degree of portal hypertension and qualify the severity of cirrhosis, Model for End-stage Liver Disease (MELD), Model for End-stage Liver Disease-Sodium (MELD-Na), Child-Turcotte-Pugh (CTP), among many, are likely not weighted properly to be meaningfully applied to ACLF specifically. Presumably due to the degree of inflammation present in ACLF, models incorporating scores detecting global organ dysfunction [SOFA, CLIF-SOFA, Acute Physiology and Chronic Health Evaluation II (APACHE II)] have been shown to be superior to liver-specific models.^20^–^22^ For example, the Chronic Liver Failure Consortium acute on chronic liver failure (CLIF-C ACLF) score has emerged as a leading prognostic model and outperformed CTP, MELD, and MELD-Na scores when predicting 28-day mortality in patients requiring admission to the hospital with ACLF. This score combines a Chronic Liver Failure—Organ Failure (CLIF-OF) score, which is a simplified addition to the aforementioned CLIF-SOFA score with age and white blood cell count.^6^,^23^ Other prognostic models have been validated in HBV-ACLF specifically.^24^–^28^ These scores have been validated only in specific cohorts (CLIF, APASL, etc.) which limits their generalizability to the overall heterogeneous global ACLF population.

The clinical course of ACLF in the initial days following the identification, that is, trajectory, likely has more prognostic value than the initial severity and might ultimately have a more meaningful impact when it comes to minimizing harm, limiting unnecessary cost, and improving end of life.^13^ EASL-CLIF ACLF grades, however, have been shown to correlate with 28-day mortality.^5^ Somewhat counter to this are data that suggest that patients with ACLF who undergo liver transplantation have similar mortality at 1 year regardless of severity grade before transplantation.^29^ Using data from the VOCAL study group evaluating patients with ACLF diagnosed using both EASL-CLIF and APASL criteria, models (VOCAL-Penn) have been developed for predicting the development of ACLF as well as mortality.^16^,^30^ They have been shown to better predict mortality when compared with MELD, MELD-Na, and CLIF-C ACLF models.^16^,^30^

### AI

**FIGURE 1:**
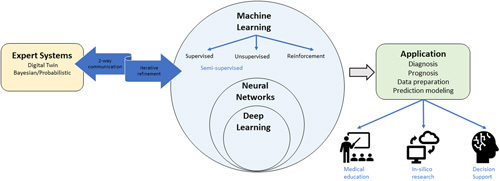
A simplified visual representation of AI applications in ACLF.

Initially labeled by John McCarthy in 1955, AI is a nondescript term used to define a broad range of techniques that allow computers to perform tasks typically thought to require human reasoning and problem-solving skills.^4^,^31^ Modern AI applications typically involve a combination of multiple techniques to achieve a desired goal. Healthcare, given its complexity, is no exception to this. What follows is an overview of a selection of AI techniques relevant to healthcare (Figure 1).

Beneath the umbrella of AI applications, machine learning (ML) encompasses various techniques including supervised ML, unsupervised ML, semisupervised learning, reinforcement learning, neural networks, and deep learning (DL). Supervised ML involves labeled input and output data which the algorithm then uses to create a mathematical model from as it “learns” relationships between input and output data. Supervised ML is a technique frequently used in healthcare. Labeled input data represent various aspects of patient-level data and labeled output data represent various desired or expected clinical endpoints. The algorithm itself interprets input data and relationships within data. Unsupervised ML allows the algorithm to extract features from unlabeled input data without output data in an exploratory nature and effectively allowing the data to “speak for itself.”^4^,^32^,^33^ Semisupervised ML, combining both supervised and unsupervised ML, incorporates unlabeled and labeled input and output data and the model is then iteratively trained as it effectively labels previously unlabeled data. Reinforcement ML models incorporate a system of reward and penalty with the algorithm targeting maximum reward or avoiding penalty based on desired outputs. These models learn iteratively by observing prior actions, sometimes in a simulated fashion, and subsequently, create and apply algorithms that maximize the return of reward-associated outputs.^34^ For simplicity’s sake, both semisupervised and reinforcement ML can also be classified as supervised ML. DL is a ML technique involving artificial neural networks (ANNs) that is designed with a number of layers between input and output data. A series of algorithms interact and form “hidden layers,” usually in a feed-forward fashion through connections that resemble neurons in a mammalian brain. The model can be further trained with more input data and errors can be addressed using a principle known as backpropagation.^31^,^35^ These models are typically developed from large and complex databases with multiple hidden neural layers and without interpretability constraints. They, therefore, provide limited transparency to the users and are described as “black-box” models. The user of “black-box” AI knows inputs and understands the outcomes of the model but how the output value was generated remains unknown due to the complexity of the algorithms created.^34^

Natural language processing is a technique that allows extraction of text from various data sources. In healthcare, this is typically used to extract text data and patient-level information which can then be utilized as input in other AI models.^4^ Knowledge engineering, more specifically expert systems, consist of designated domain experts contributing known or consensus-derived knowledge to better understand complex human decisions or outline complex human physiology or pathophysiology. Bayesian or probabilistic models, particularly in the form of directed-acyclic graphs (DAGs), use variables and their conditional dependencies to create a visually accessible way of representing expert systems and causal pathways. Multiple less complex DAGs can then be combined into larger, more complex hierarchical models.^36^ Digital twins are simulation models, created using DAGs using both data and expert knowledge, which can receive inputs from their real-world “twin” and return outputs that can enhance insight and assist in decision-making.^34^ Digital twins have been developed in various engineering and geopolitical arenas but are only recently being applied to healthcare.^37^–^39^ These models offer the benefit of being able to provide actionable knowledge whether in an educational environment, in research, or at the bedside.^34^

### AI and ACLF

In recent years, there has been a significant increase in the application of AI to the fields of critical care and hepatology. This has coincided with significant advancement in AI methodology and computing power as well as the availability of large data cohorts. A recent review by Ahn et al^4^ summarizes various applications of AI in hepatology. Prior prognostic modeling and scoring tools for ACLF utilized logistic regression and related methodology. It is important to note, however, that the majority of these and other models have been developed in a data-driven fashion utilizing registry data or retrospective cohorts.^4^,^40^ These models tend to evaluate long-term outcomes and in some cases improve diagnosis, but they may only provide limited prognostic enrichment compared with prior models which mostly provided long-term prognostic estimates. Interestingly, when used for clinical modeling various ML approaches had previously been shown to be no better than traditional statistical approaches, but this field is growing rapidly.^41^

A few recent studies in ACLF patients have effectively utilized AI approaches. Table 2 outlines prognostic models and other approaches leveraging AI methods recently applied to ACLF.^42^–^46^

**TABLE 2:** AI models in ACLF

References	Methodology	Criteria	Outcome	Comparators	Performance	Comments
Zheng et al^42^	ANN	APASL	90-d mortality	MELD, MELD-Na, MELDNa, MESO, iMELD	AUROC 0.765, *p* < 0.0001	Causes of ACLF other than HBV were excluded
Xu et al^43^	Imbalanced and semisupervised ML, SVM		Predictive accuracy	MELD		Model designed to predict outcomes in cohorts with missing or incomplete data
Shi et al^24^	CART, LR	APASL (suspected)	90-d mortality	MELD	AUROC CART: 0.896, LR 0.914 *p* < 0.001	Causes of ACLF other than HBV were excluded
Garcia et al^44^	Semisupervised ML, LR	EASL-CLIF	Predictive accuracy for ACLF-related mortality			Methodological approach to data preparation for AI modeling
Hou et al^45^	ANN	APASL	28- and 90-d mortality	MELD, MELD-Na, CTP, CLIF-ACLF	AUROC 0.748 and 0.754, respectively	Causes of ACLF other than HBV were excluded
Musunuri et al^46^	ANN	APASL	30- and 90-d mortality		AUROC 0.915 and 0.921, respectively	Patients with malignancy were excluded

Abbreviations: ACLF, acute on chronic liver failure; AI, artificial intelligence; ANN, artificial neural network; APASL, Asian Pacific Association for the Study of the Liver; AUROC, area under the receiver operating characteristic; CART, classification and regression tree; CTP, Child-Turcotte-Pugh; EASL-CLIF, European Association for the Study of the Liver—Chronic Liver Failure; iMELD, incorporating serum sodium and age Model for End-stage Liver Disease model; LR, logistic regression; MELD, Model for End-stage Liver Disease; MESO, Model for End-stage Liver Disease to serum sodium ratio; ML, machine learning; SVM, support vector machine.

Zheng and colleagues created and validated a model including patients diagnosed with HBV-associated ACLF by APASL criteria. Patients with causes of liver disease other than HBV were excluded from both the training and validation cohorts. Using ANN, patient-level modeling was compared with various MELD-based predictors of mortality with 90-day mortality as an outcome. In the validation cohort, authors reported an area under the receiver operating characteristic 0.765 (95% CI: 0.608–0.722, *p* < 0.0001) demonstrating increased accuracy compared with common MELD-based scoring systems.^42^ Hou and colleagues created and validated 2 ANN models to predict 28- and 90-day mortality also in patients with HBV-associated ACLF meeting APASL criteria. Compared with MELD, both models were found to be more accurate using the training and validation data sets. This study was novel given the creation of an accurate 28-day mortality prediction model using ML. There were differences in predictive accuracy between the training and validation cohorts for both models that may be explained by the varied mortality rates in each cohort.^45^ Musunuri and colleagues similarly created and validated an ANN that unlike prior models included numerous predictive scores such as MELD, quickSOFA, CLIF-SOFA, and CTP in addition to demographic and biochemical characteristics. Patients included were diagnosed with HBV-associated ACLF by APASL criteria. The validated models demonstrated predictive accuracy for 30- and 90-day mortality with the area under the receiver operating characteristic 0.915 and 0.921, respectively.^46^

Shi and colleagues evaluated a large cohort of over 1000 patients with suspected ALCF due to HBV and created and validated novel classification and regression tree and logistic regression models that outperformed the traditional MELD score with the area under the receiver operating characteristic 0.896 and 0.914, respectively (*p* < 0.001). Given their improved accuracy and specificity to ACLF, these scoring systems may serve as comparators for future ML approaches, especially when evaluating 90-day mortality in patients with ACLF by APASL criteria.^24^

In a methodological approach, Xu and colleagues utilized supervised learning techniques similar to small sphere and large margin and twin support vector machines to develop prediction models for ACLF patients with missing demographic and biochemical data and imbalanced mortality and survival outcome data. Models were then compared based on their ability to accurately predict mortality. Mortality prediction was then also correlated with MELD scores.^43^ Garcia and colleagues utilized the large CANONIC database consisting of patients meeting EASL-CLIF criteria for ACLF in a methodologic and exploratory study. The authors outlined logistic regression-related methods for preparing heterogeneous patient data with many inconsistencies ranging from missing data resulting from patient death and high short-term mortality to large amounts of noninformative patient-level data. They targeted patient characteristics expected to predict mortality and formulated an approach to preparing such data for ML application to be used in future studies.^44^ML techniques have also been used to predict liver transplant outcomes including allograft fibrosis and while these were not targeted specifically toward an ACLF population they may ultimately be applicable when combined with other AI applications.^47^–^51^

These studies demonstrate the benefits of utilizing AI approaches for predictive modeling and other aspects of ACLF. When compared with traditional scoring systems there was improved prognostic accuracy. These approaches present tools for preparing and analyzing heterogeneous or incomplete data, which are common for ACLF patient-level data, with higher accuracy than the currently applied standards which were not created for ACLF patients. However, with the exception of the methodologic study conducted by Garcia and colleagues which included patients meeting EASL-CLIF criteria for ACLF, data to date are limited in scope to a predominantly HBV-associated ACLF cohort fulfilling APASL criteria. Further, the majority of the predictive models developed using AI approaches utilized an ANN which is a black-box approach. In the context of ACLF, AI approaches are especially subject to limitations related to such black-box approaches, biases in dataset creation, limited and heterogeneous data due to high short-term mortality, preexisting geographical cohort distributions, and minimal prospective data available for validation.

When thinking about the near future, applying novel AI techniques to ACLF patient populations has the potential to shed light on treatment strategies, transform our current understanding of patient outcomes including organ transplantation, and even highlight previously unknown or misunderstood physiological and pathophysiological mechanisms.^52^ ML, more specifically reinforcement learning, has been shown to “learn” optimal treatment strategies in septic patients which represent a population with a complex and incompletely understood pathophysiology similar to ACLF.^53^ AI, specifically supervised ML, offers the opportunity to improve on existing prognostic and predictive models in ACLF and the potential to significantly impact disease trajectory in the short term. There may also be benefit to ML approaches to ACLF populations to better understand pathophysiology, adapt or redefine current definitions, outline novel phenotypes, or influence prospective trial design.^54^–^58^

To date, we are unaware of data evaluating natural language processing or digital twins and other expert systems in patients with ACLF but data have demonstrated that expert systems can replicate and accurately estimate outcomes in diseases with complex underlying pathophysiology such as diabetes and sepsis.^59^–^61^ These data are promising given similarities to ACLF given the acute on chronic flow of patients throughout health systems similar to diabetes and complex pathophysiological mechanisms outlined in ACLF resembling sepsis.^9^ DAGs, which are developed from current understanding of the pathophysiology of disease are forms of expert systems that one can envision will allow one to identify points (variables) where interventions may have impact on disease progress and therefore impact outcomes through clinical decision support. With an iterative approach using expert consensus, retrospective and prospective data, digital twins can be iteratively refined, validated, and avoid or limit the downsides of traditional “black-box” ML approaches.^36^ These “in-silico” patients offer a means to assess interventions at or at least temporally proximate to the point of care. Providers can then assess expected impact without exposing actual patients to interventions that are not so useful or even harmful. One can also envision a more personalized approach to care of ACLF patients by leveraging AI such as natural language processing, ML, and other relevant data to create digital twins or other expert systems and tailor therapies to each patient throughout various phases of their care.

We anticipate that eventually these models can also be used for medical education and other training purposes and, with proper validation, to evaluate decisions in critically ill patients including those with ACLF without exposing patients to unnecessary risk.

### Ethical considerations and AI limitations

While AI, and, more specifically, expert systems will enable a more individualized approach to care, we must be mindful of ethical issues and address them before and as they arise. In North America and Europe, major oversight organizations have set regulations on AI applications but such applications in healthcare are a new phenomenon. With the rapid expansion of AI in healthcare, regulatory mechanisms remain limited in their scope especially when it comes to ML applications.^62^

Data privacy, including individual and institutional rights to health-related data, will require consideration. Availability of these technologies could also have ethical and societal implications due to discrimination and isolation of certain populations especially when it comes to the potential for training validation data sets with bias and subsequent resource allocation or decision support. As these technologies are developed, proper refinement and transparency will be required and during this process they should augment and supplement provider decision-making rather than replace it. Inevitably, decisions “influenced” by AI will have implications when it comes to allocation of responsibility and lines between algorithm-based decisions and clinician decisions will become further blurred. Questions regarding well-established ethical concepts such as patient autonomy, beneficence, nonmaleficence, and justice will inevitably arise.^37^,^63^

## CONCLUSIONS

ACLF is a recently established syndrome associated with significant mortality. Current data are almost exclusively limited to predictive and prognostic models developed using traditional statistical approaches based on retrospective data. Modern AI approaches are increasingly being applied to ACLF and those previously developed for similarly complex disease processes can likely be applied to ACLF to enhance education and training as well as to aid decision-making. As with all new technologies and especially given the complexity of related decision-making, ethical implications, and methodologic considerations unique to these new approaches must be considered when applying AI to patients with ACLF.
